# Nerve decompression and neuropathy complications in diabetes: Are attitudes discordant with evidence?

**DOI:** 10.1080/2000625X.2017.1367209

**Published:** 2017-09-06

**Authors:** D. Scott Nickerson

**Affiliations:** ^a^ Northeast Wyoming Wound Care Center, Sheridan, WY, USA

**Keywords:** Nerve decompression, diabetic neuropathy and complications, treatment of diabetic neuropathy complications, diabetic foot ulcer prevention, diabetic foot ulcer economics

## Abstract

External neurolysis of the nerve at fibro-osseous tunnels has been proprosed to treat or prevent signs, symptoms, and complications in the lower extremity of diabetes patients with sensorimotor polyneuropathy. Nerve decompression is justified in the presence of symptomatic compressed nerves in the several fibro-osseous tunnels of the extremities, which are known to be frequent in diabetes. Quite a body of literature has accumulated reporting results after such nerve decompression in the leg, describing pain relief and sensibility improvement, as well as balance recovery, diabetic foot ulcer prevention, curtailed ulcer recurrence risk, and amputation avoidance.

Historical academic hesitance to endorse surgical treatments for pain and numbness in diabetes was based primarily on the early retrospective reports’ potential for bias and placebo effects, and that the hypothetical basis for surgery lies outside the traditional etiology paradigm of length-dependent axonopathy. This reticence is here critiqued in view of recent studies using objective, measured outcome protocols which nullify such potential confounders. Pain relief is now confirmed with Level 1 studies, and Level 2 prospective information suggests protection from initial diabetic foot ulceration and most neuropathic ulcer recurrences. In view of the potential for nerve decompression to be useful in addressing some of the more difficult, expensive, and life altering complications of diabetic neuropathy, this secondary compression thesis and operative treatment methodology may deserve reassessment.

## Introduction

The use of operative nerve decompression (ND) in treating diabetic neuropathy symptoms and complications was suggested nearly 30 years ago. ND has never been widely adopted, and proponents and skeptics hold differing strong opinion about its appropriate use. A recent systematic review of diabetic foot ulcer (DFU) treatments [1] and fresh guidance on ulcer prevention from the International Working Group on the Diabetic Foot (IWGDF) both now mention ND [2]. A review of published evidence about this external neurolysis technique in diabetes is timely and appropriate.

In 1988, an *Annals of Plastic Surgery* editorial [3] suggested that there was reason to be optimistic about treating the symptoms and complications of diabetic neuropathy. Based upon animal laboratory experimentation, the author postulated that metabolic effects of diabetes mellitus (DM) caused measureable physical nerve enlargement, resulting secondarily in focal nerve trunk compressions at fibro-osseous tunnels which serve as peri-articular anatomic anchoring points. These entrapments and impingements might then result in areas of local conduction block which could be identified clinically by Tinel’s percussion test, and would respond to relief of compression via operative external neurolysis. Dellon’s [4] 1992 article on clinical results of such surgical ND for 154 compressed nerves in patients with diabetic sensorimotor polyneuropathy (DSPN) reported improved motor and/or sensory function in most, while progressive deterioration continued in contralateral limbs not operated on. The suggestion was made that ‘symptoms of sensorimotor diabetic neuropathy may be due partly to compression of multiple peripheral nerves’, and the results ‘further suggest that surgical decompression of such nerves may result in symptomatic improvement’.

A number of subsequent reports [5–10] corroborated that ND could apparently be beneficial for the neuropathic pain which is a primary concern of DSPN patients, and also for the sensibility loss [9] which enables complications like DFU and its frequent grim sequelae of infection, sepsis, gangrene, amputation, and early mortality.

The idea that surgical treatment could be of significant benefit in a metabolic disease was challenging to the status quo, and not embraced by most clinicians or academicians caring for diabetes and DSPN complications. With the ND approach originating in the surgical sub-specialities, out of the mainstream of endocrinology, neurology, and diabetes neuropathy treatment and research, criticism of this option was common and committed. Critiques were at times vehement, and sometimes personal, with unfortunate implications that the procedure might be fraudulent and primarily driven by surgeon economic self-interest. A polemic commentary by Cornblath et al. [11] best presented the concerns of the skeptical neurology and endocrinology academic community. Assessments of the evidence were published as a clinical advisory by the American Academy of Neurology (AAN) and a Cochrane Review, with Chaudhry being lead author of both [12,13], and yielding a pronouncement that this approach was rated ‘unproven’ scientifically.

Both Cornblath et al. and Chaudhry et al. strongly recommended further research to test ND by strong science, but have not themselves been involved in such efforts. Pending such results, they advised that the ND option be neither presented nor recommended as an alternative to DSPN patients. A decade has now passed since those Cornblath critiques were issued. So it is timely to review the published, peer reviewed scientific evidence suggesting that DSPN might involve more than a metabolically determined LDA.

## The skeptics case

The Cornblath et al. [11] commentary is Level 5 evidence, expert opinion devoid of new experimental evidence. They asserted the ND procedure was being utilized for treating symptomatic and generalized DSPN based on the following ‘questionable hypotheses’, which they assigned to ND surgery proponents:

1. The signs and symptoms of DSPN are due to multiple nerve entrapments.
2. The entrapments can be diagnosed by a trained examiner using only the Tinel Sign.
3. Surgical ‘release’ of these nerves will ‘correct’ DSPN.
4. Special surgical training is required to diagnose and operate on this condition.

Proponents view these assertions as incomplete unless qualified thus:

1. DSPN is a metabolically mediated neuropathy, with *secondary* nerve entrapments commonly found and causally related in varying degrees to symptoms, signs, and sequelae.
2. After diagnosing DSPN by clinical examination and elimination of other neuropathy diagnoses, the Tinel sign indicates areas of nerve regeneration or hyper-reactivity, and allows one to clinically select candidates most likely to benefit from operative ND.
3. Surgical ND by external neurolysis can improve the signs and symptoms related to the secondary nerve entrapments and appear to significantly relieve pain and protect against DSPN foot complications.
4. Safe and effective ND procedures require surgeon familiarity with peripheral nerve anatomy, its variability, and use of safe microsurgical techniques. If this knowledge has not been acquired in residency training, it should be learned from experienced instructors and include anatomic dissections.

Cornblath et al. [11] lament that these hypotheses have ‘spawned an entire industry’. They enumerate nine issues at odds with current DSPN dogma and the presumptive etiology known as length dependant axonopathy (LDA). These are listed below (with comments in italics on their validity and pertinence):

1. In DSPN, progressive distal axonal loss occurs, as do sensory and motor signs and symptoms proximal to the anatomic entrapment sites. *This point, even if accurate, is not dispositive of the ND thesis. Metabolically based secondary local nerve entrapment could co-exist with LDA in DSPN.*
2. Frequency of peripheral nerve entrapments in diabetes is small. *Point 2 is baldly false, and co-author Vinik had published a 30% prevalence for nerve entrapments in diabetes, hardly a ‘small’ incidence* [14].
3. The diagnostic Tinel sign is not standardized, was initially described to indicate nerve regeneration rather than entrapment, lacks sensitivity, and is non-specific. *Again, the critique is not dispositive. Tinel’s sign has proven clinically useful as a predictor of ND benefit* [15,16].
4. Published ND reports rank as mostly low grade evidence, from uncontrolled, unblinded, retrospective cohort studies. *True in 2006, but Level 2* [17,18] *and Level 1* [19–20] *evidence is now available. Not dispositive. Low level evidence, although scientifically weaker, is not ergo false, just as Cornblath et al.’s level 5 EBM commentary need not be correct.*
5. An AAN practice advisory and a Cochrane Review article [5,6] grade ND as of Level U, or unproven utility for symptomatic DSPN. *True, but not dispositive. Both call for better science. Subsequent Level 1 evidence of pain relief is compelling*.
6. Immediate pain relief in the recovery suite, bilateral pain improvement after unilateral surgery, and 80–92% of patients reporting relief were too astounding to believe, and no hypothesis for such an unexpected finding was proferred [17,21–25]. *Not dispositive. The bilateral pain relieving effect of unilateral decompression is confirmed in two Level 1 studies* [19], *although the mechanism is unknown. A retrospective study demonstrates intra-op EMG motor function improvement during ND* [26]. *RCT protocols confirm high expectation of pain relief with ND in comparison to disappointing benefits with pharmacology.*
7. These treatments were being promoted to patients and could be a large expense to the medical system. *This is speculative opinion, no more, and not dispositive. One-time surgery cost and durable pain relief seen with ND compares favorably with $1000/year expense of ongoing pharmacologic treatment for seriously painful DSPN* [27].
8. Surgical and non-surgical interventions for other conditions have sometimes failed to fulfill their early promise. *Very weak point. Not dispositive, and of unknown relevance to the ND issue.*
9. Bias, placebo effects, and the natural history of DSPN might explain the hopeful reports of pain relief after ND in the setting of DSPN. *Speculation. Level 1 RCT studies have shown this conjecture to be false* [19].

This is quite a list of caveats! However, reflecting upon of their relevance, one must acknowledge that points 1, 3, 4, 5, 6, 7, 8, and 9 do not dispose of the hypothesis. By this, we mean they do not exclude a nerve entrapment involvement in DSPN symptoms and complications. Points 1, 3, 5, 6, 7, and 8 might be factual, but are immaterial to the validity of the entrapment and compression hypothesis. Point 6 highlights subjective clinical observations [10] inconsistent with LDA, for which no etiopathogenic hypothesis then existed, but have subsequently been confirmed. Point 7 is nothing more than speculation. Point 8 makes no statement about ND. Only points 4 and 9 are pertinent observations which can be scientifically tested for validation or nullification of the nerve compression hypothesis.

## Published evidence

Several laboratory animal studies support the importance of nerve compression in diabetes [28,29]. Streptozotocin-induced diabetes in rats produces sciatic nerve swelling. Compression induced by a sciatic-encircling latex tube altered the walking trackway appearance, and produced mechanical allodynia with concomitant demyelination of spinal cord afferents  and diminished GABAbeta levels in dorsal horn neurons. The trackway alteration can be prevented with pre-disease ND of the tarsal tunnel analogue, and the GABAbeta levels reconstituted by ND which removes the encircling latex tube [30].

Human clinical ND studies have used both subjective and objective outcome measures. Subjective outcomes are based upon patient report, so could indeed be subject to bias and placebo effects. Relief of neuropathic pain and recovery of protective sensation, which are of primary patient interest, provide scientifically weak information unless prospective, randomized control protocols are used. However, objectively measured outcomes, like electrophysiology recordings, measured ulceration and recurrence risk, perineural tissue pressures, measured circulatory changes, or balance and stability performance could invalidate such critiques. The following catalog of published ND reports specifies outcomes measured, their objectivity or subjectivity, and pertinent references to the DSPN sign, symptom, or complication assessed.

## Subjective outcomes

### Pain and sensation, retrospective

Pain is often measured with the 11-point Likert Visual Analog Scale (VAS), ranking increasing pain levels from 0–10. Touch sensation is measured with the Semmes-Weinstein monofilaments (SWM), 2-point Discriminator, or the Pressure Specified Sensory Device (PSSD), which is a painless, non-invasive, semi-objective device. The PSSD measures pressure levels at which a sensory input is perceived and reported by the subject.

Pain relief occurs in around 80% of ND cases according to two meta analyses [31,32]. Valdivia et al. [10] confirmed similar retrospective results at 1 year in over 100 cases. Liao et al. [33] found ND effective in both diffuse and focal DSPN pain for 300 cases with 2 year F/U vs a control, non-operated group. Anderson et al. [26] found strong pain relief in a study of intra-operative EMG changes during ND.

### Pain and sensation, prospective

Sensibility usually improves after ND, while contralateral control sites show progressive loss [7,9]. Gondring et al. [34] showed SWM monofilament evaluation of plantar sensation improved with tarsal tunnel ND, as did Mazilu et al. [35]. Zhang et al. [25] found post-op recovery of two-point sensibility to nearly normal levels. The level 1 EBM randomized control trial of Macare van Maurik et al. [19] found lasting pain relief in operated limbs at 1 year, in comparison to the contralateral control limbs, which interestingly also improved and is pertinent to Cornblath et al.’s [11] point 6.

### Tinel sign

Gentle fingertip percussion of nerve trunks at anatomic entrapment sites is prognostic of pain relief if it produces a tingling sensation distally or proximally. This positive Tinel sign is common in DSPN and correlates strongly with the Michigan Neuropathy Screening Instrument [16]. Pain relief after ND is found in 80% if Tinel’s sign at anatomic entrapment sites is positive, but only 50% if negative [15,16,36].

### Touch sensibility

In 15 mostly retrospective studies, some restitution of protective sensibility occurs in 80% of cases [37]. The Zhang et al. [25] prospective study reports recovery of hallux pulp 2 point sensation from > 9 mm to near normal levels at mean 6.7 mm, in 560 advanced DSPN and DFU cases. Liao et al. [33] confirmed this for both hallux and digiti minimi with a controlled, prospective study.

### Vibration sensibility, prospective

Vibration perception threshold (VPT) is improved significantly by ND [25,35,38].

### Quantitative sensory testing, prospective

Both cold and warm temperature perception improve after nerve decompression [25,38].

### Skin ichthyosis resolution

Anecdotal reports of improved skin tone and resolution of ichthyosis exist, suggesting sympathetic system skin effects, but such results have not been published in any study.

## Objective outcomes

### Resolution of tarsal tunnel conduction block

**Figure 1.**
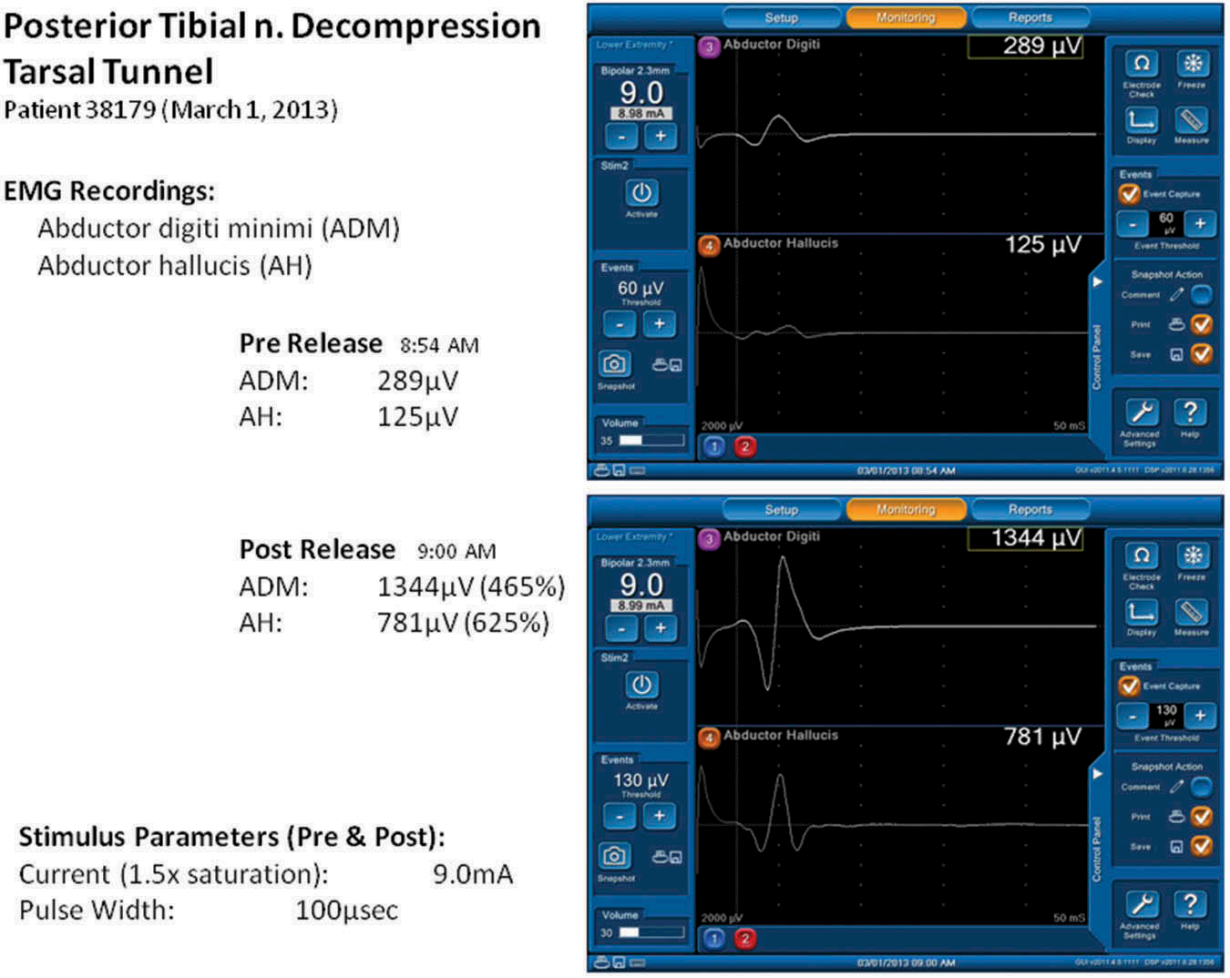
NIM screenshot figure of 400% improvement of motor evoked potential in the 6-minute interval for ND of posterior tibial and plantar nerves.

Anderson and Barrett et al. [26,39] demonstrate acute improvement in evoked EMG potential in some DSPN cases within 1 minute of surgical external neurolysis for decompression of tarsal tunnel, medial and lateral plantar tunnels, as Figure 1 illustrates.

### Common peroneal nerve (CPN) conduction block changes

Zhang et al. [40] report over 70% of diabetics had leg nerve conduction abnormalities, with CPN being most commonly involved. Acute intra-operative improvement in EMG motor evoked potentials of anterior and lateral compartment leg muscle can occur with decompression of the common peroneal nerve at the fibular neck [26,39]. Stimulation of CPN proximal to the fibular tunnel is followed by improved motor evoked potential response of peroneus longus and tibialis anterior after ND in most cases.

### Nerve trunk enlargement in DSPN

This is a key tenet of the Dellon nerve compression hypothesis. A number of reports document such enlargement is present in DSPN, as measured by ultrasonography [25,38,41–44] and MRI [45].

### Decrease in post-operative nerve cross-sectional area by ultrasonography

Zhang et al. [25], Zhong et al. [38], Liao et al. [33], and Macare van Maurik et al. [42] all report reduced size of nerve after ND. The latter series found the non-operated contralateral control leg nerve was also smaller, to a similar degree. This and a similar contralateral pain benefit [19,20] in unilateral ND are not understood, but have been reported multiple times.

### Nerve conduction velocity (NCV) change in DSPN

Confirming Zhang et al. [46] and Zhong et al. [38], Rota et al. [47] report NCV changes typical of entrapment neuropathy to be present in 70% of a consecutive series of patients referred to an Italian diabetology unit.

### Nerve conduction velocity (NCV) recovery post-ND

Recovery of diminished pre-op NCV and maintenance at 18 months after ND has been observed [25,38,39]. However, Macare van Maurik et al. [48] found no change at 12 months by a differing technique.

### Visualization of constricted nerve at fibro-osseous tunnel

**Figure 2.**
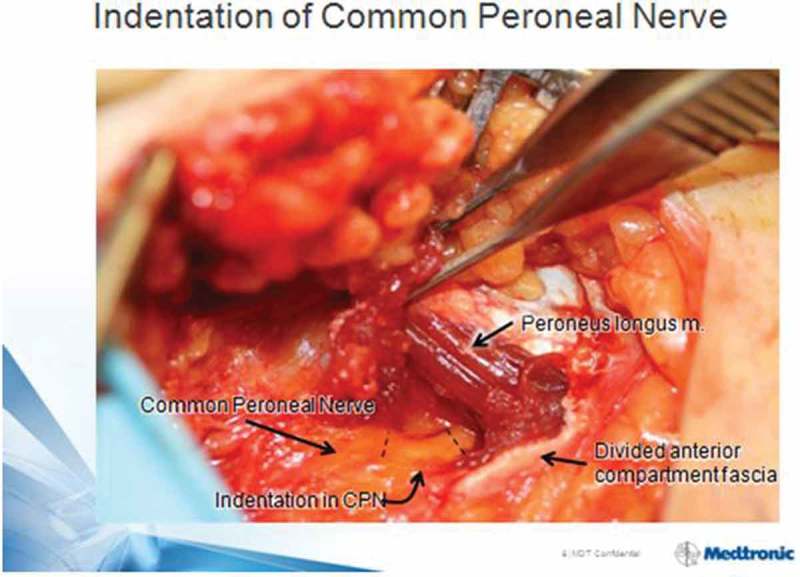
Indentation of common peroneal nerve at R fibular neck is noted just after decompression by division of peroneus longus fascia and a few muscle fibers. Magnification = 1.5×. Patella at 12 o’clock direction, foot to 4:30. With permission of Dr. S. Barrett, Phoenix, AZ.

The intra-operative appearance of deep peroneal nerve in the dorsal foot and common peroneal nerve at fibular neck often demonstrate focal narrowing or indentations, as in Figure 2, which visually resolve in the first minutes after ND [37,39].

### Toe clawing resolution, retrospective

Reversal of toe clawing due to intrinsic foot muscle atrophy or paralysis in DSPN has been observed post-ND [49].

### Post-ND infection risk, retrospective

ND cases in a registry do not experience the expected 10% or higher infection risk which Wukich [24,25,50] has reported for foot surgeries in diabetes cases complicated by DSPN.

### Primary ulceration risk, prospective

Protection against primary diabetic foot ulcer (DFU) and amputation in the medium term has been demonstrated with ND for advanced DSPN [21,25,33].

### DFU recurrence or amputation risk, retrospective and prospective

**Figure 3.**
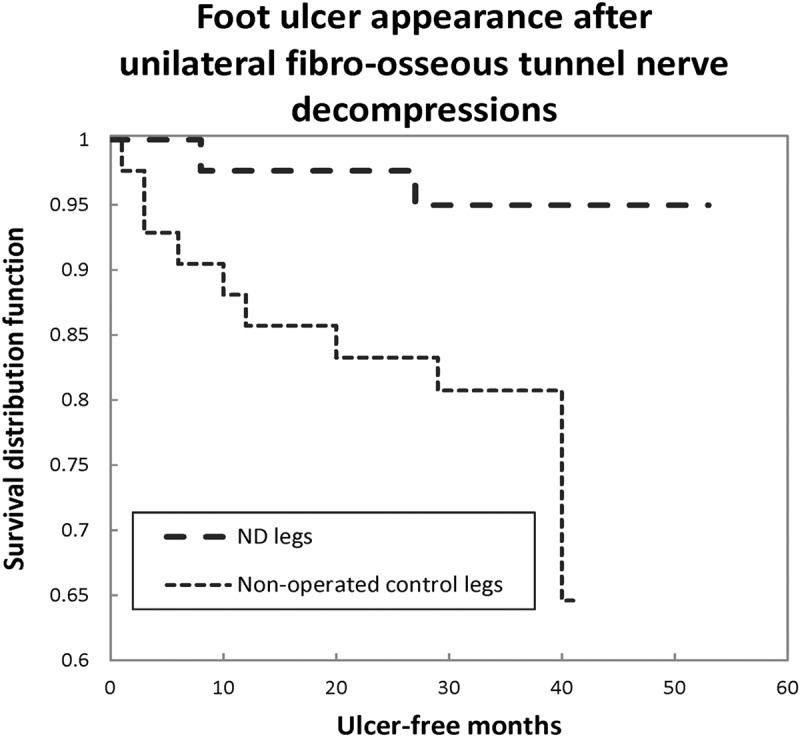
A Kaplan Meier survival curve illustrates the ulcer-free survival of 42 cases with prior healed unilateral DFU and subsequent ND of that leg only. The previously intact contralateral leg, without ND, has a relative risk of subsequent ulceration of 5.5 (*p *= 0.048). From Nickerson and Rader, JAPMA 104:66–70 (2014), with permission.

Nerve decompression appears to reduce the documented 25–30% annual DFU recurrence risk [51] by 80% or more [17,22–26], Figure 3 demonstrates one report. Trignano et al [52] reported 100% healing and 0/8 recurrences of neuro-ischemic DFU at 18 months after ND.

### Healing of recalcitrant or indolent ulcerations

Anecdotal reports suggest that ND may trigger healing of the recalcitrant or indolent DFU. Only one case report exists at present [53].

### Hospitalizations for infection, retrospective

The risk of hospitalization for foot infection in diabetes, at 0.6%/yr in a registry of 628 ND cases with painful DSPN [15], is much lower after ND than the 2.4%/yr reported by Lavery et al. [54].

### Neuroischemia by TcpO2 and ultrasound, prospective

Trignano et al. [52] found 40 diabetic feet with ischemic levels of transcutaneous skin oxygenation (TcpO2) below 40 mm Hg had returned to levels above 40 after ND in 90% of cases by month 3 and 95% by 18 months. Nominal improvement occurred in 100%. Eight pre-operative ulcers healed and none had occurred post-operatively at 18 months after ND. Improvements were maintained or improved further from 3–18 months post-surgery in 36 of 40 limbs and decreased by 1 mm Hg in four. Tekin et al. [55] report ND procedures have a positive effect on ultrasonic hemodynamic and morphological parameters (increased pulsatility and decreased resistance) of the dorsalis pedis and posterior tibial arteries.

### Balance and stability prospectively measured

Balance, as measured by tilt table, improves for diabetics to normal for age when bilateral ND, but not unilateral ND, is performed [56,57]. The latter Macare van Maurik et al. [57] paper, using only unilateral ND, unfortunately muddles the situation by stating that there is no evidence that ND in the leg affects stability in painful DSPN within 1 year. Their study tests only the effect of single leg ND, equivalent to testing depth perception after restoring sight to one eye in the blind.

### High peri-neural tissue pressure, prospective

In DSPN with secondary nerve entrapment, tissue pressures in the peri-neural area as measured by the Weck catheter method are elevated to dangerous levels of 25 mm Hg. Such levels are expected to affect vasa nervorum circulation and neural axoplasmic flow. With ND, intra-operative pressures return to normal levels under 10 mm [18].

## Meta-analyses and reviews

### Meta-analysis

Dellon [37] reviewed pain and sensibility results, finding pain relief in 88%, and improved sensation in 79% from 15 reports. The Baltodano et al. [31] review found ND to be strongly effective for pain relief in 91%, and improved sensibility in 69%, as well as protecting against ulceration and amputations (Odds ratio = 0.066. *p* < 0.0001).

### Systematic analysis

Van Netten et al. [1], in their comprehensive review on prevention of DFU in the at-risk patient, included ND in their summary of surgical methods, stating, ‘Surgical interventions can be effective in selected patients, but the evidence base is small’.

### IWGDF guidance

Bus et al. [2] recommend: ‘Do not use a nerve decompression procedure in an effort to prevent a foot ulcer in an at-risk patient with diabetes, in preference to accepted standards of good quality care’. This guidance is a weak strength recommendation, based upon low quality of evidence.

### Decision tree analysis

Garrod et al. [58] used a decision-tree analytic approach to achieve an efficacy estimate for medical management versus surgical decompression in patients presenting with diabetic neuropathy and chronic superimposed tibial and peroneal nerve compressions. Based upon the available evidence from the literature, this model demonstrates the potential advantage of the strategy of decompression surgery over medical management for ulceration (*p* = < 0.002) and LEAP amputation prevention (*p* = < 0.001).

### Economic cost modeling

Rankin et al. [27] illustrate the potential benefit of applying ND to healed neuropathic DFU, which could quickly yield USA cost reductions in the range of $1 billion annually by prevention of ulcer recurrences. Hyperspectral imaging technology exists to prospectively identify advanced DSPN cases who will soon develop an ulceration, offering the prospect of further savings by using ND to avoid the primary DFU [59]. Raising TcpO2 in neuro-ischemic situations suggests further opportunity for avoiding DFU cases and costs [52].

## Discussion

A number of findings from the objective outcome protocols seem particularly interesting. Demonstration of relief of high perineural pressure, improved balance stability with bilateral leg neurolysis, and protection from neuropathic DFU ulcer and recurrence are of tremendous import clinically. The restitution of skin oxygenation in neuro-ischemic feet gives further reason for optimism about minimizing foot complications. DSPN patients are all concerned about balance, falls and fractures, and the justifiable fear of the loss to amputation of their legs, liberty, and mobility. Studies demonstrating medium-term improvement in NCV and intra-operative EMG values are quite suggestive of both physiologic and clinical benefit, despite the fact that ND ameliorates only focal nerve compression and entrapment. Full restoration of neural function is, nevertheless, precluded by the primary metabolic effects.

Behavioral, functional, biochemical, and ultrastructural alterations in nerves and neurons are known both in animal diabetes models and chronic nerve compression laboratory studies, giving perspective on the human clinical results. Both intra-neural sorbitol accumulation and oxidative stress linked to metabolic flux via aldose reductase are thought to be important in DSPN [60], although aldose reductase targeting drugs have generally been disappointing clinically.

Nerve size changes would play a role in producing the hypothesized focal compressions addressed by ND. Post-operative change in caliber of the decompressed nerve trunks has been variously reported and denied. Implications and interpretations can be theorized for either result. On the one hand focal compression has been relieved and axoplasmic pressures theoretically normalized. Yet the hyperglycemia, inducing sorbitol hyperosmolarity, would remain to induce persisting intra-neural fluid flow effects.

Dyck et al. [61,62] reported that, although conduction block and diminished vibratory sensibility are induced by mechanical compression in diabetic animal nerves there is apparent protection against axonal degeneration, and suggested the frequent entrapment neuropathy in diabetes might be due to pathologic changes not in the nerve, but in fibro-osseous structures. They reported compression-induced lengthening of axonal internodes, obscuration of nodes of Ranvier, and widening of internodal gaps. Such microstructural changes might produce nerve action potential instability and a lowered threshold for discharge linked to lancinating pains and allodynia. Neural ischemia in diabetes is well established with diminished production and function of endothelium-derived vasodilators like nitric oxide, and exaggerated production of vasoconstrictors, leading to endothelial dysfunction and elevated vascular tone culminating in macro- and microvascular damage. Basement membrane thickening, pericyte degeneration, and endothelial cell hyperplasia in endoneurial microvessels strongly correlate with clinical defects and nerve pathology. Human and animal models have shown endoneurial hypoxia caused by a reduction in nerve blood flow and increased endoneurial vascular resistance. There is strong evidence that ischemia is crucial in DSPN nerve fiber degeneration and loss [63]. Yet, Jaramillo et al. [64] notes resistance of the action potential to inactivation by the neural ischemia observed in diabetic patients, allowing continued function at reduced levels.

Investigations by the UC Irvine orthopedic group [65,66] have confirmed in mice that compression causes demyelination/remyelination that can be relieved by neurolysis, most effective if done early, as Siemionow et al. [67] and Liao et al. [33] have shown clinically. Schwann cells can respond directly to mechanical stimuli, such as nerve gliding through a fibro-osseous tunnel, by proliferating and down-regulating myelin-associated proteins like myoinositin, while desert hedgehog protein limits demyelination extent [66,68]. Axonal sprouting without Wallerian degeneration is seen histologically at compression sites. Pham and Gupta [69] lead us to think that chronic nerve compression injury is a Schwann cell-mediated disease.

Further indication of the key role played by focal nerve trunk compression in diabetes is the ability of tarsal tunnel analog ND in diabetic rats to prevent or reverse neuropathic walking track patterns and amplify withdrawal from painful stimulus [70,71]. Pain reductions after ND also imply that peripheral generation of pain by focal nerve compression is important, perhaps dominant in DSPN, and also that substantial centralization of neuropathic pain is not universal. If central sensitization were dominant, ND could not produce relief so frequently.

Several factors may be involved in academic resistance to the possibility that ND could be useful in DSPN. Rarely do non-surgical clinicians have the opportunity to personally observe the yellowish discoloration of enlarged, edematous peripheral nerves of diabetes shown in Figure 2. Second, Dellon’s ND theory stands outside the currently accepted hypothesis of length-dependent axonopathy as DSPN etiopathogenesis. Third, early ND proponents sometimes carelessly spoke of it as a treatment for neuropathy, failing to strongly emphasize its usefulness was specifically, and only, for the secondary nerve entrapment symptoms now known to be so commonly associated with the DSPN process [72].

Regarding the AANS and Cochrane reviews, injudicious skeptics and critics have misquoted the ‘unknown’ conclusion as ‘ineffective’ or ‘not recommended’, which is specious. The weakness of ND scientific evidence pointed out by Chaudhry et al. [12,13] was that most reports at the time were retrospective cohort studies using subjective outcome measures. Such protocols are indeed subject to risks of placebo effect and bias of patient, observer, or surgeon. To address this shortcoming, skeptical critics suggested that only prospective studies with randomized controls, blinded observers, and sham surgery protocols could provide evidence which might prove ND effective. This unfortunately would put sham study patients at the same > 10% risk of peri-operative infectious complication which has been raised as another objection to common usage of ND, and would confront any institutional review board asked to grant study approval.

Nonetheless, such a study, including Sham surgery, has now been accomplished by Rozen et al. [] and presented twice at a conference and at the 2017 ADA Scientific Sessions, demonstrating strong Level 1 evidence of gratifying and durable pain relief. Its imminent publication should provide the indisputable peer-reviewed evidence of valuable ND benefit which is required for ND to achieve wide acceptance.

So, what observations might be made in light of available evidence? First, confirmation has accumulated of clinical benefit in use of ND to treat signs and symptoms of DSPN. The scientific strength of this evidence has progressed from only case reports and retrospective clinical reviews to include Level 2 prospective studies and some Level 1 evidence. Still, expert opinion continues to be divided. To contradict the proponents of ND, the skeptics seem to rely heavily on the established evidence that axonopathy exists. This axonopathy is evidenced by clinical, laboratory, imaging, and histological evidence, and is not in dispute. This does not preclude related, co-existant, superimposed compression and entrapment effects. Skeptics misinterpret ND proponents to be indicating that nerve compression is the sole cause of DSPN, a claim which has never been made. The Dellon hypothesis clearly posits that nerve entrapment is a secondary, but significant, factor in producing DSPN signs, symptoms, and complications. Hence, ND is appropriate only for the limited painful group, who can be identified as having nerve trunk entrapment and have not found relief with other measures. The value of applying ND to DSPN complication groups such as neuropathic or neuro-ischemic ulcers is robustly suggested by clinical evidence, but not yet tested with Level 1 RCT studies.

Strong protocols using measureable objective outcomes have offered an opportunity to resolve the ND dilemma with strong science. Objective outcome measure protocols rather than subjective patient pain and sensibility assessments are not subject to the placebo and bias confounders and should yield scientifically strong results without requiring blinding or sham procedures. Studies examining the post-ND risk of DFU occurrence, recurrence, or subsequent amputations are good examples. Azsmann [3] presented the first report of ND affecting subsequent foot complications, finding that, in DSPN, patients operated on only *unilaterally* for pain, 30% developed DFU or underwent amputation over the subsequent 5 years. Each and every complication event in 50 subjects occurred in the contralateral, non-operated leg (*p* = 0.001). At least six studies have confirmed ND can provide significant, although not total protection against DFU and recurrence [17,21–25]. Studies also exist using as outcomes perineural tissue pressure [18], transcutaneous oxygen pressures [37], and measured balance [56,72].

The depth and growing strength of data and observations outlined here seem discordant with continuing skepticism of ND, and ought to encourage an urgent re-evaluation of ND attitudes. The ‘nays’ consider the matter closed based on the decade-old Cornblath critique, whose limited relevance we have scrutinized. The ‘ayes’ have been gathering stronger evidence of ND value in various DSPN situations. In two new papers, the IWGDF has considered ND reports for the first time in a systematic review and guidance for preventing foot ulcers in at-risk diabetes patients [1,2]. It may now be an appropriate time for our thought leaders to re-evaluate the possibility that ND has significant promise for DSPN.

Science needs to be skeptical, and requires evidence to amend or improve the functioning paradigm, in this case LDA alone being causal of DSPN. Skeptical ongoing reappraisal is the path to progress. Theoretical physicist Albert Einstein observed: ‘Everyone sits in the prison of his own ideas; he must burst it open’ (Albert Einstein, Herbert Spencer lecture delivered at Oxford, Jun. 10, 1933 Read more at http://notable-quotes.com/e/einstein_albert_ii.html#aGXlkOcVop516Viy.99). So, skeptics must periodically re-evaluate published data, and supply more than only defiant opinion and enduring opposition. To do otherwise is certainly not in the interest of the millions in the USA, let alone the world, who suffer from formidable DSPN complications, and look hopefully to medicine for answers. The panoply of published observations and evidence strongly suggests that length dependant axonopathy cannot fully explain DSPN and a place must be recognized for multiple peripheral nerve entrapments. A new more inclusive paradigm recognizing the contribution of nerve entrapments both explains clinical findings more completely and offers, as Dellon suggested in 1988, 'a cause for optimism in diabetic neuropathy' [3]. Whatever further studies, protocols, and investigations are lacking, in the opinion of some, to further test the value of this apparently effective and protective therapy, let them be defined and begin at once. I believe we owe this to our patients.

## References

[1] Van Netten JJ, Price PE, Lavery LA, et al Prevention of foot ulcers in the at-risk patient with diabetes: a systematic review. Diabetes Metab Res Rev. 2016;32(Suppl 1):84–11. 26340966 10.1002/dmrr.2701

[2] Bus SA, Van Netten JJ, Lavery LA, et al IWGDF guidance on the prevention of foot ulcers in at-risk patients with diabetes. Diabetes Metab Res Rev. 2016;32(Suppl 1):16–24. 26334001 10.1002/dmrr.2696

[3] Dellon AL. A cause for optimism in diabetic neuropathy. Ann Plast Surg. 1988;20(2):103–105. 3355053 10.1097/00000637-198802000-00001

[4] Dellon AL Treatment of symptomatic diabetic neuropathy by surgical decompression of multiple peripheral nerves. Plast Reconstr Surg. 1992;89(4):689-697; discussion 698-689. 1546082

[5] Wood WA, Wood MA, Werter SA, et al Testing for loss of protective sensation in patients with foot ulceration: a cross-sectional study. J Am Podiatr Med Assoc. 2005;95(5):469–474. 16166466 10.7547/0950469

[6] Wieman TJ, Patel VG Treatment of hyperesthetic neuropathic pain in diabetics. Decompression of the tarsal tunnel. Ann Surg. 1995;221(6):660-664; discussion 664–665. 10.1097/00000658-199506000-00005 PMC1234690 7794070

[7] Rader AJ Surgical decompression in lower-extremity diabetic peripheral neuropathy. J Am Podiatr Med Assoc. 2005;95(5):446–450. 16166461 10.7547/0950446

[8] Biddinger KR, Amend KJ The role of surgical decompression for diabetic neuropathy. Foot Ankle Clin. 2004;9(2):239–254. 15165580 10.1016/j.fcl.2003.12.001

[9] Aszmann OC, Kress KM, Dellon AL Results of decompression of peripheral nerves in diabetics: a prospective, blinded study. Plast Reconstr Surg. 2000;106(4):816–822. 11007394 10.1097/00006534-200009040-00010

[10] Valdivia Valdivia JM, Weinand M, Maloney CT Jr., et al Surgical treatment of superimposed, lower extremity, peripheral nerve entrapments with diabetic and idiopathic neuropathy. Ann Plast Surg. 2013;70(6):675–679. 23673565 10.1097/SAP.0b013e3182764fb0

[11] Cornblath DR, Vinik A, Feldman E, et al Surgical decompression for diabetic sensorimotor polyneuropathy. Diabetes Care. 2007;30(2):421–422. 17259523 10.2337/dc06-2324

[12] Chaudhry V, Stevens JC, Kincaid J, et al Practice advisory: utility of surgical decompression for treatment of diabetic neuropathy: report of the therapeutics and technology assessment subcommittee of the american academy of neurology. Neurology. 2006;66(12):1805–1808. 16801641 10.1212/01.wnl.0000219631.89207.a9

[13] Chaudhry V, Russell J, Belzberg A Decompressive surgery of lower limbs for symmetrical diabetic peripheral neuropathy. Cochrane Database Syst Rev. 2008;3:CD006152. 10.1002/14651858.CD006152.pub2 PMC8990523 18646138

[14] Vinik A, Mehrabyan A, Colen L, et al Focal entrapment neuropathies in diabetes. Diabetes Care. 2004;27(7):1783–1788. 15220266 10.2337/diacare.27.7.1783

[15] Dellon AL, Muse VL, Scott ND, et al A positive Tinel sign as predictor of pain relief or sensory recovery after decompression of chronic tibial nerve compression in patients with diabetic neuropathy. J Reconstr Microsurg. 2012;28(4):235–240. 22411625 10.1055/s-0032-1306371

[16] Shar Hashemi S, Cheikh I, Lee Dellon A Prevalence of upper and lower extremity tinel signs in diabetics: cross-sectional study from a USA, urban hospital-based population. J Diabetes Metab. 2013;4(245):2.

[17] Nickerson DS, Rader AJ Nerve decompression after diabetic foot ulceration may protect against recurrence: a 3-year controlled, prospective analysis. J Am Podiatr Med Assoc. 2014;104(1):66–70. 24504579 10.7547/0003-0538-104.1.66

[18] Rosson GD, Larson AR, Williams EH, et al Tibial nerve decompression in patients with tarsal tunnel syndrome: pressures in the tarsal, medial plantar, and lateral plantar tunnels. Plast Reconstr Surg. 2009;124(4):1202–1210. 19935304 10.1097/PRS.0b013e3181b5a3c3

[19] Macare Van Maurik JF, van Hal M, van Eijk RP, et al Value of surgical decompression of compressed nerves in the lower extremity in patients with painful diabetic neuropathy: a randomized controlled trial. Plast Reconstr Surg. 2014;134(2):325–332. 24732651 10.1097/PRS.0000000000000369

[20] Rozen S, Wolfe G, Raskin P, et al DNND (Diabetic Neuropathy Nerve Decompression) study - a controlled, randomized, double-blinded, prospective study on the role of surgical decompression of lower extremity nerves for the treatment of patients with symptomatic diabetic neuropathy with chronic nerve compression. In: 77th American Diabetes Association Scientific Sessions 2017; San Diego (CA).

[21] Aszmann O, Tassler PL, Dellon AL Changing the natural history of diabetic neuropathy: incidence of ulcer/amputation in the contralateral limb of patients with a unilateral nerve decompression procedure. Ann Plast Surg. 2004;53(6):517–522. 15602245 10.1097/01.sap.0000143605.60384.4e

[22] Nickerson DS Low recurrence rate of diabetic foot ulcer after nerve decompression. J Am Podiatr Med Assoc. 2010;100(2):111–115. 20237362 10.7547/1000111

[23] Nickerson DS, Rader AJ Low long-term risk of foot ulcer recurrence after nerve decompression in a diabetes neuropathy cohort. J Am Podiatr Med Assoc. 2013;103(5):380–386. 24072366 10.7547/1030380

[24] Dellon AL, Muse VL, Nickerson DS, et al Prevention of ulceration, amputation, and reduction of hospitalization: outcomes of a prospective multicenter trial of tibial neurolysis in patients with diabetic neuropathy. J Reconstr Microsurg. 2012;28(4):241–246. 22411624 10.1055/s-0032-1306372

[25] Zhang W, Zhong W, Yang M, et al Evaluation of the clinical efficacy of multiple lower-extremity nerve decompression in diabetic peripheral neuropathy. Br J Neurosurg. 2013;27(6):795–799. 23713665 10.3109/02688697.2013.798854

[26] Anderson JC, Nickerson DS, Tracy BL, et al Acute improvement in intraoperative EMG following common fibular nerve decompression in patients with symptomatic diabetic sensorimotor peripheral neuropathy: 1. EMG results. J Neurol Surg A Cent Eur Neurosurg. 2017;78(5):419–430. 10.1055/s-0036-1593958 28038479

[27] Rankin TM, Miller JD, Gruessner AC, et al. Illustration of cost saving implications of lower extremity nerve decompression to prevent recurrence of diabetic foot ulceration. J Diabetes Sci Technol. J Neurol Surg A Cent Eur Neurosurg. 2017;78(5):419–430. doi:10.1055/s-0036-1593958. PMC4525647 26055081

[28] Dellon AL, Dellon ES, Seiler WAt. Effect of tarsal tunnel decompression in the streptozotocin-induced diabetic rat. Microsurgery. 1994;15(4):265–268. doi:10.1002/(ISSN)1098-2752 8035673

[29] Kale B, Yuksel F, Celikoz B, et al Effect of various nerve decompression procedures on the functions of distal limbs in streptozotocin-induced diabetic rats: further optimism in diabetic neuropathy. Plastic And Reconstructive Surgery. 2003;111(7):2265-2272. doi:10.1097/01.PRS.0000060100.80687.D9 12794469

[30] Liao CYM, Zhong W, Liu P, et al Association of myelinated primary afferents impairment with mechanical allodynia in diabetic peripheral neuropathy: an experimental study in rats Oncotarget. 2017; https://doi.org/10.18632/oncotarget.19359 10.18632/oncotarget.19359 PMC5609991 28969059

[31] PA, Basdag B Bailey CR, et al. The positive effect of neurolysis on diabetic patients with compressed nerves of the lower extremities: a systematic review and meta-analysis. Plast Reconstr Surg Glob Open. J Diabetes Sci Technol. 2015;9(4):873–880. doi:10.1177/1932296815584796. PMC4173835 25289218

[32] Dellon AL The Dellon approach to neurolysis in the neuropathy patient with chronic nerve compression. *Handchirurgie, Mikrochirurgie, plastische Chirurgie: Organ der Deutschsprachigen Arbeitsgemeinschaft fur Handchirurgie: Organ der Deutschsprachigen Arbeitsgemeinschaft fur Mikrochirurgie der Peripheren Nerven und Gefasse*. 2008;40(6):351–360. 10.1055/s-2008-1039216 19051159

[33] Liao C, Zhang W, Yang M, et al Surgical decompression of painful diabetic peripheral neuropathy: the role of pain distribution. PloS One. 2014;9(10):e109827. 25290338 10.1371/journal.pone.0109827 PMC4188608

[34] Gondring WH, Tarun PK, Trepman E Touch pressure and sensory density after tarsal tunnel release in diabetic neuropathy. Foot Ankle Surg. 2012;18(4):241–246. 23093118 10.1016/j.fas.2012.02.001

[35] Mazilu G, Budurca RA, Graur M, et al [Surgical treatment of tarsal tunnel syndrome in diabetic neuropathy]. Rev Med Chir Soc Med Nat Iasi. 2012;116(1):128–134. 23077884

[36] Lee CH, Dellon AL Prognostic ability of Tinel sign in determining outcome for decompression surgery in diabetic and nondiabetic neuropathy. Ann Plast Surg. 2004;53(6):523–527. 15602246 10.1097/01.sap.0000141379.55618.87

[37] Dellon AL Neurosurgical prevention of ulceration and amputation by decompression of lower extremity peripheral nerves in diabetic neuropathy: update 2006. Acta Neurochir. 2007;100:149–151. 10.1007/978-3-211-72958-8_32 17985566

[38] Zhong W, Zhang W, Yang M, et al Impact of diabetes mellitus duration on effect of lower extremity nerve decompression in 1,526 diabetic peripheral neuropathy patients. Acta Neurochirurgica. 2014;156(7):1329–1333. 24760499 10.1007/s00701-014-2087-8

[39] Barrett S, Levine T, Hank N, et al A pilot trial of peripheral nerve decompression for painful diabetic neuropathy. In: American academy of neurology convention 2013 3 16–23; San Diego (CA).

[40] Zhang W, Li S, Zheng X Evaluation of the clinical efficacy of multiple lower extremity nerve decompression in diabetic peripheral neuropathy. J Neurol Surg A Cent Eur Neurosurg. 2013;74(2):96–100. 23250876 10.1055/s-0032-1320029

[41] Lee D, Dauphinee DM Morphological and functional changes in the diabetic peripheral nerve: using diagnostic ultrasound and neurosensory testing to select candidates for nerve decompression. J Am Podiatr Med Assoc. 2005;95(5):433–437. 16166459 10.7547/0950433

[42] Macare Van Maurik JF, Schouten ME, Ten Katen I, et al Ultrasound findings after surgical decompression of the tarsal tunnel in patients with painful diabetic polyneuropathy: a prospective randomized study. Diabetes Care. 2014;37(3):767–772. 24379356 10.2337/dc13-1787

[43] Riazi S, Bril V, Perkins BA, et al Can ultrasound of the tibial nerve detect diabetic peripheral neuropathy? A cross-sectional study. Diabetes Care. 2012;35(12):2575–2579. 23033242 10.2337/dc12-0739 PMC3507587

[44] Breiner A, Qrimli M, Ebadi H, et al Peripheral nerve high-resolution ultrasound in diabetes. Muscle Nerve. 2017;55(2):171–178. 27312883 10.1002/mus.25223

[45] Thakkar RS, Del Grande F, Thawait GK, et al Spectrum of high-resolution MRI findings in diabetic neuropathy. AJR Am J Roentgenol. 2012;199(2):407–412. 22826404 10.2214/AJR.11.7893

[46] Zhang Y, Li J, Wang T, et al Amplitude of sensory nerve action potential in early stage diabetic peripheral neuropathy: an analysis of 500 cases. Neural Regen Res. 2014;9(14):1389–1394. 25221597 10.4103/1673-5374.137593 PMC4160871

[47] Rota E, Zavaroni D, Parietti L, et al Ulnar entrapment neuropathy in patients with type 2 diabetes mellitus: an electrodiagnostic study. Diabetes Res Clin Pract. 2014;104(1):73–78. 24565211 10.1016/j.diabres.2014.01.024

[48] Macare van Maurik JF, Franssen H, Dw M, et al Nerve conduction studies after decompression in painful diabetic polyneuropathy. J Clin Neurophysiol. 2015;32(3):247–250. 26035677 10.1097/WNP.0000000000000169

[49] Dellon AL, Steck JK Reversal of toe clawing in the patient with neuropathy by neurolysis of the distal tibial nerve. Microsurgery. 2008;28(5):303–305. 18537170 10.1002/micr.20513

[50] Wukich DK, Crim BE, Frykberg RG, et al. Neuropathy and poorly controlled diabetes increase the rate of surgical site infection after foot and ankle surgery. the Journal Of Bone & Joint surgery. 2014;96(10):832–839. doi:10.2106/JBJS.L.01302. 24875024 PMC4018772

[51] Lavery LA, La Fontaine J, Kim PJ Preventing the first or recurrent ulcers. Med Clin North Am. 2013;97(5):807–820. 23992893 10.1016/j.mcna.2013.05.001

[52] Trignano E, Fallico N, Chen HC, et al Evaluation of peripheral microcirculation improvement of foot after tarsal tunnel release in diabetic patients by transcutaneous oximetry. Microsurgery. 2016;36(1):37–41. doi:10.1002/micr.22378. 25641727

[53] Trignano E, Fallico N, Zingone G, et al Combined treatment of diabetic foot ulcer with tarsal tunnel release and perilesional injections of peripheral blood mononuclear cells. J Am Podiatr Med Assoc. 2017;107(2):171–174. 28394682 10.7547/15-098

[54] Lavery LA, Wunderlich RP, Tredwell JL Disease management for the diabetic foot: effectiveness of a diabetic foot prevention program to reduce amputations and hospitalizations. Diabetes Res Clin Pract. 2005;70(1):31–37. 16126121 10.1016/j.diabres.2005.02.010

[55] Tekin F, Agladioglu K, Surmeli M, et al The ultrasonographic evaluation of hemodynamic changes in patients with diabetic polyneuropathy after tarsal tunnel decompression. Microsurgery. 2015;35(6):457–462. 26235134 10.1002/micr.22467

[56] Ducic I, Taylor NS, Dellon AL Relationship between peripheral nerve decompression and gain of pedal sensibility and balance in patients with peripheral neuropathy. Ann Plast Surg. 2006;56(2):145–150. 16432321 10.1097/01.sap.0000194246.18332.23

[57] Macare Van Maurik JF, Ter Horst B, Van Hal M, et al Effect of surgical decompression of nerves in the lower extremity in patients with painful diabetic polyneuropathy on stability: a randomized controlled trial. Clin Rehabil. 2015;29(10):994–1001. doi:10.1177/0269215514556298. 25381348

[58] Garrod KLAS, McCabe S, Lee DA Prevention of ulceration and amputation, by neurolysis of peripheral nerves in diabetics with neuropathy and nerve compression: decision-tree utility analysis. J Diabetes Metab. 2014;5(1):5.

[59] Yudovsky D, Nouvong A, Schomacker K, et al Assessing diabetic foot ulcer development risk with hyperspectral tissue oximetry. J Biomed Opt. 2011;16(2):026009. 21361693 10.1117/1.3535592 PMC3048880

[60] Oates PJ Aldose reductase, still a compelling target for diabetic neuropathy. Curr Drug Targets. 2008;9(1):14–36. 18220710 10.2174/138945008783431781

[61] Dyck PJ, Engelstad JK, Giannini C, et al Resistance to axonal degeneration after nerve compression in experimental diabetes. Proc Natl Acad Sci U S A. 1989;86(6):2103–2106. 2928319 10.1073/pnas.86.6.2103 PMC286856

[62] Dyck PJ, Lais AC, Giannini C, et al Structural alterations of nerve during cuff compression. Proc Natl Acad Sci U S A. 1990;87(24):9828–9832. 2263633 10.1073/pnas.87.24.9828 PMC55267

[63] Nukada H Ischemia and diabetic neuropathy. Handb Clin Neurol. 2014;126:469–487. 25410240 10.1016/B978-0-444-53480-4.00023-0

[64] Jaramillo J, Simard-Duquesne N, Dvornik D Resistance of the diabetic rat nerve to ischemic inactivation. Can J Physiol Pharmacol. 1985;63(7):773–777. 3930062 10.1139/y85-128

[65] Gupta R, Rowshan K, Chao T, et al Chronic nerve compression induces local demyelination and remyelination in a rat model of carpal tunnel syndrome. Exp Neurol. 2004;187(2):500–508. 15144876 10.1016/j.expneurol.2004.02.009

[66] Jung J, Hahn P, Choi B, et al Early surgical decompression restores neurovascular blood flow and ischemic parameters in an in vivo animal model of nerve compression injury. J Bone Joint Surg Am. 2014;96(11):897–906. 24897737 10.2106/JBJS.M.01116 PMC4049242

[67] Siemionow M, Sari A, Demir Y Effect of early nerve release on the progression of neuropathy in diabetic rats. Ann Plast Surg. 2007;59(1):102–108. 17589271 10.1097/01.sap.0000252067.95690.9b

[68] Tapadia M, Mozaffar T, Gupta R Compressive neuropathies of the upper extremity: update on pathophysiology, classification, and electrodiagnostic findings. J Hand Surg Am. 2010;35(4):668–677. 20223605 10.1016/j.jhsa.2010.01.007 PMC4715364

[69] Pham K, Gupta R Understanding the mechanisms of entrapment neuropathies. Review article. Neurosurg Focus. 2009;26(2):E7. 10.3171/FOC.2009.26.2.E7 19435447

[70] Barac S, Jiga LP, Barac B, et al Hindpaw withdrawal from a painful thermal stimulus after sciatic nerve compression and decompression in the diabetic rat. J Reconstr Microsurg. 2013;29(1):63–66. 23161393 10.1055/s-0032-1328917

[71] Dellon AL, Dellon ES, Seiler W Effect of tarsal tunnel decompression in the streptozotocin-induced diabetic rat. Microsurgery. 1994;15(4):265–268. 8035673 10.1002/micr.1920150409

[72] Vinik AI Diabetic neuropathy: pathogenesis and therapy. Am J Med. 1999;107(2b):17s–26s. 10484041 10.1016/s0002-9343(99)00009-1

[73] Ducic I, Short KW, Dellon AL Relationship between loss of pedal sensibility, balance, and falls in patients with peripheral neuropathy. Ann Plast Surg. 2004;52(6):535–540. 15166971 10.1097/01.sap.0000122654.65588.f0

